# Engineering plant membranes using droplet interface bilayers

**DOI:** 10.1063/1.4979045

**Published:** 2017-03-23

**Authors:** N. E. Barlow, E. Smpokou, M. S. Friddin, R. Macey, I. R. Gould, C. Turnbull, A. J. Flemming, N. J. Brooks, O. Ces, L. M. C. Barter

**Affiliations:** 1 Department of Chemistry, Imperial College London, Exhibition Road, South Kensington, London SW7 2AZ, United Kingdom; 2 Institute of Chemical Biology, Imperial College London, Exhibition Road, South Kensington, London SW7 2AZ, United Kingdom; 3 Department of Life Sciences, Imperial College London, Sir Alexander Fleming Building, South Kensington SW7 2AZ, United Kingdom; 4 Syngenta, Jealott's Hill International Research Centre, Bracknell, Berkshire RG42 6EY, United Kingdom

## Abstract

Droplet interface bilayers (DIBs) have become widely recognised as a robust platform for constructing model membranes and are emerging as a key technology for the bottom-up assembly of synthetic cell-like and tissue-like structures. DIBs are formed when lipid-monolayer coated water droplets are brought together inside a well of oil, which is excluded from the interface as the DIB forms. The unique features of the system, compared to traditional approaches (e.g., supported lipid bilayers, black lipid membranes, and liposomes), is the ability to engineer multi-layered bilayer networks by connecting multiple droplets together in 3D, and the capability to impart bilayer asymmetry freely within these droplet architectures by supplying droplets with different lipids. Yet despite these achievements, one potential limitation of the technology is that DIBs formed from biologically relevant components have not been well studied. This could limit the reach of the platform to biological systems where bilayer composition and asymmetry are understood to play a key role. Herein, we address this issue by reporting the assembly of asymmetric DIBs designed to replicate the plasma membrane compositions of three different plant species; *Arabidopsis thaliana*, tobacco, and oats, by engineering vesicles with different amounts of plant phospholipids, sterols and cerebrosides for the first time. We show that vesicles made from our plant lipid formulations are stable and can be used to assemble asymmetric plant DIBs. We verify this using a bilayer permeation assay, from which we extract values for absolute effective bilayer permeation and bilayer stability. Our results confirm that stable DIBs can be assembled from our plant membrane mimics and could lead to new approaches for assembling model systems to study membrane translocation and to screen new agrochemicals in plants.

## I. INTRODUCTION

**FIG. 1.**
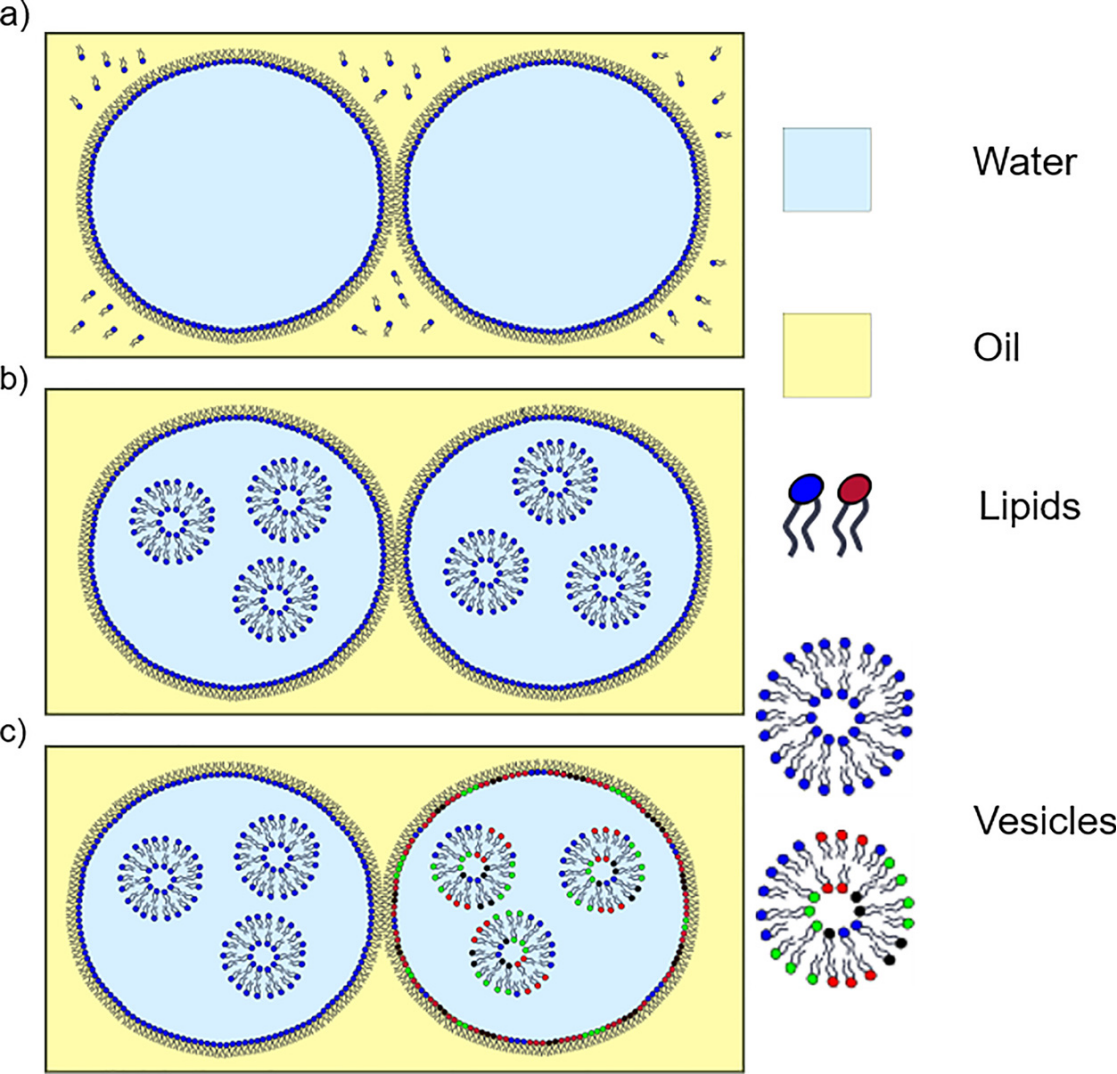
Schematic diagram showing the assembly of droplet interface bilayers in different operating modes. (a) In the lipid-out approach, lipids are added directly to the oil, whereas in (b) the lipid-in approach, lipid vesicles are supplied to the water droplets. (c) Asymmetric DIBs can be engineered by supplying droplets with different vesicles as illustrated. In each case, the lipids supplied to the system assemble to form a monolayer around each droplet and a DIB is formed when the droplets are manipulated into contact. The number of lipid species (indicated by the different colors) in part (c) is to reflect the complex compositions of our plant lipid preparations.

Droplet interface bilayers (DIBs) are model membranes formed when lipid-monolayer coated water droplets are manipulated into contact inside a well of oil.^1,2^ The method works either by adding lipids directly to the oil using the lipid-out method (Fig. 1(a)) or by supplying lipids to the droplets in the form of vesicles with the lipid-in approach (Fig. 1(b)). In each case, a lipid-monolayer self-assembles at the oil/water interface and a DIB is formed when the droplets are manipulated into contact, which can be achieved in a number of ways including manual or robotic droplet anchors,^3,4^ electric fields,^5^ optical traps,^6^ compressible substrates,^7^ via magnetic beads,^8^ or using droplet microfluidic systems.^9,10^

DIBs can be engineered to a prescribed size and composition,^7,11^ used to perform electrical measurements of reconstituted ion channels,^3,12,13^ and employed to study the translocation of small molecules across membranes. The key advantages of DIBs compared to supported lipid bilayers, black lipid membranes, and liposomes are that they are easy to form,^1^ offer high stability,^14,15^ can be used to assemble asymmetric bilayers by supplying different lipids to each water droplet (Fig. 1(c)),^6,10,16^ and can be assembled into multi-component bilayer networks consisting of up to thousands of microdroplets.^17,18^ These attributes, coupled with the ability to control interdroplet communication by functionalizing selected membranes inside an array with protein pores,^18,19^ have led DIBs to become increasingly regarded as powerful minimal tissue constructs. Yet a key drawback of the DIB platform is that the compositions of the bilayers assembled are typically oversimplified and are not representative of biological membranes, where the relationship between lipid composition and membrane function is understood to play a key role. This limitation presents a bottleneck to the applicability of DIBs to biological systems, such as plant membranes, where representative lipid compositions are vital for studying the tightly regulated function of intracellular nanochannels such as plasmodesmata,^20,21^ and for uncovering the biological engineering rules that regulate and control the efficient translocation of endogenous proteins, small molecules, or agrochemicals^22^ across plant membranes.

Here, we report the assembly of asymmetric DIBs designed to replicate the plasma membranes of *Arabidopsis thaliana*,^23^ tobacco,^24^ and oats^25^ by controlling the relative amount of phospholipid, phytosterol, and sphingolipid present in the bilayer. We used a Soy Polar Lipid Extract (SPLE) to provide the most abundant phospholipids present in a typical plant membrane^26–29^ and plant phytosterols to control the plasma membrane fluidity and permeability. We performed a systematic study to demonstrate that stable vesicles can be made from our different plant membrane mimics and show that each of these vesicle populations can be used to assemble asymmetric DIBs (as illustrated schematically in Fig. 1(c)), which we verify for each condition by measuring effective bilayer permeation. Our findings suggest that our approach could be scaled to assemble higher-order networks of model plant DIBs to study the translocation of endogenous proteins, small molecules, and agrochemicals in plants.

## II. MATERIALS AND METHODS

### A. Phospholipid and vesicle preparation

All lipids were purchased from Avanti Polar Lipids and dissolved in chloroform according to the phospholipid-sterol-cerebroside (PSC) ratios outlined in Table I. Lipid films were generated by evaporating the chloroform under a stream of nitrogen and were subsequently dried by placing inside a vacuum desiccator for at least 1 h. Vesicles were made by resuspending the lipid film in buffer (100 mM KCl, 10 mM HEPES, pH 7.3), subjecting the dispersion to 5 freeze-thaw cycles and extruding by passing 21 times through polycarbonate membrane with an average pore size of 100 nm.^10^ The total amount of lipid used in each preparation was 10 mg/ml. SPLE has a phospholipid profile (wt/wt) of 45.7% phosphatidylcholine (PC), 22.1% phosphatidylethanolamine (PE), 18.4% phosphatidylinositol (PI), and 6.9% phosphatidic acid (PA) with the remaining 6.9% unknown.

**TABLE I.** Lipid compositions of model plant DIBs (mol. %).

Sample	SPLE	Stigmasterol	Sitosterol	Glucocerebroside
PSC 1 (*Arabidopsis thaliana*)	50	20	20	10
PSC 2 (Tobacco)	42	15	15	28
PSC 3 (Oats)	44	7	7	42

### B. Calcein release assay

Calcein leakage assays were performed as described by Powl *et al*.^30^ Vesicles were prepared at a concentration of 6.25 mg/ml in a buffer containing 20 mM HEPES, 100 mM KCl, and 50 mM calcein, pH 7.4. To remove unencapsulated calcein dye, 300 *μ*l of the vesicle dispersion was passed through a Sephadex G-25 column (GE Healthcare). The column was washed and eluted with buffer (20 mM HEPES, 100 mM KCl, 0.5 M Sucrose, pH 7.4) until collections were faintly orange in colour. The leakage assay was performed with 200 *μ*l samples on a 96-well plate using a Fluorescence Spectrophotometer (Varian). A baseline was recorded during 0–60 min and 120–180 min with an excitation wavelength at 490 nm and an emission wavelength of 520 nm. After 180 min, the vesicles were ruptured by the addition of 1 *μ*l of 0.2 M octaethylene glycol monododecyl ether solution in sucrose buffer. The immediate rise in fluorescence intensity was monitored for a further 30 min.

### C. Device fabrication

Separate platforms were engineered for imaging individual DIBs or DIB networks using brightfield microscopy, and for assembling arrays of DIBs for performing the bilayer permeation assay. For brightfield microcopy, 150 *μ*m deep wells (ø = 1.2 mm) were fabricated from Polydimethylsiloxane (PDMS) (Sylgard 184, Dow Corning) using standard soft lithography techniques. Briefly, a 150 *μ*m thick layer of SU-8 2050 (MicroChem) was deposited on a 4″ silicon wafer and patterned with a photomask (Micro Lithography Services Ltd.) under UV light. The patterned SU-8 film was developed in Microposit™ EC solvent (Dow) before the wafer was cleaned, dried, and silanized under vacuum. PDMS mixed to a ratio of 10:1 (base:curing agent) was then poured onto the mask, degassed, and baked overnight at 65 °C. The cured PDMS wells were diced with a scalpel and plasma bonded (Plasma Cleaner Model PDC 002, Harrick Plasma) to another piece of PDMS containing a large well to allow the micro-wells to be completely submerged in oil. For the bilayer permeation assay, 5 × 5 circular wells (ø = 1.2 mm) were engineered from 0.2 mm thick PMMA (Weatherall) using a flatbed laser cutter (Universal Systems). The wells were adhered to a glass slide with silicon oil painted on the edges and an additional frame was similarly fixed to the top surface of the chip to maximize the oil coverage of the wells.

### D. Bilayer formation and characterization

DIBs were formed by completely filling the respective devices with hexadecane and dispensing 0.6 *μ*l (PDMS wells) or 0.1 *μ*l (PMMA wells) of vesicles in buffer using a pipette. After a 2 min incubation period, the droplets were gently pushed into contact with a needle and imaged using a microscope. The images were subsequently analyzed using a MATLAB image processing script to extract droplet dimensions including volume and surface area, as well as relative fluorescence intensity as previously demonstrated.^31^ Axial symmetry is assumed with respect to the interface length, which allows the area to be estimated directly as a function of interface radius. The radius is defined as half of the linear distance between the points of the circle-circle intersection.

### E. Effective bilayer permeation assay

To study the effective bilayer permeation, the top row of the 5 × 5 PMMA device was loaded with calibration droplets containing 10, 5, 2.5, 1.25, and 0.63 *μ*M resorufin, while the remaining 20 wells were used to assemble DIBs. Fluorescence intensity measurements were taken every 5–10 min for up to an hour with a Typhoon FLA 7000 (GE Healthcare) laser scanner (set to a 25 *μ*m pixel resolution) equipped with a TAMRA filter. A MATLAB script was written using the Image Processing Toolbox to find droplet geometric centres and measure the average fluorescence intensity within each droplet. The effective permeability $\;P\;[\text{cm}\;s^{-1}]$ is measured as a function of diffusion rate $\;k\left[s^{-1}\right]$, droplet volume $\;V\left[{\text{cm}}^{3}\right]$, and interfacial area $\;A\left[{\text{cm}}^{2}\right]$, $P=\frac{\text{kV}}{A}$. The diffusion rate can be found by least squares fitting of the empirical data of the normalised fluorescence intensity ${\left[I\right]}_{i}$ of droplet  i in Equation (1)

$${\left[I\right]}_{i}=c_{i}\;e^{-2kt}+\frac{1}{2};\;{\left[I\right]}_{i_{0}}-\frac{1}{2}=c_{i}.$$ (1)

This technique, as applied previously,^31^ infers first order rates of diffusion, an assumption used widely for membrane permeability of small molecules.^32–34^ Due to the Unstirred Water Layer (UWL) effect, the actual membrane permeability will be an underestimate from the actual intrinsic membrane permeability and must be interpreted as relative values not absolute.^35,36^

## III. RESULTS AND DISCUSSION

### A. Vesicle formation from plant lipids

**FIG. 2.**
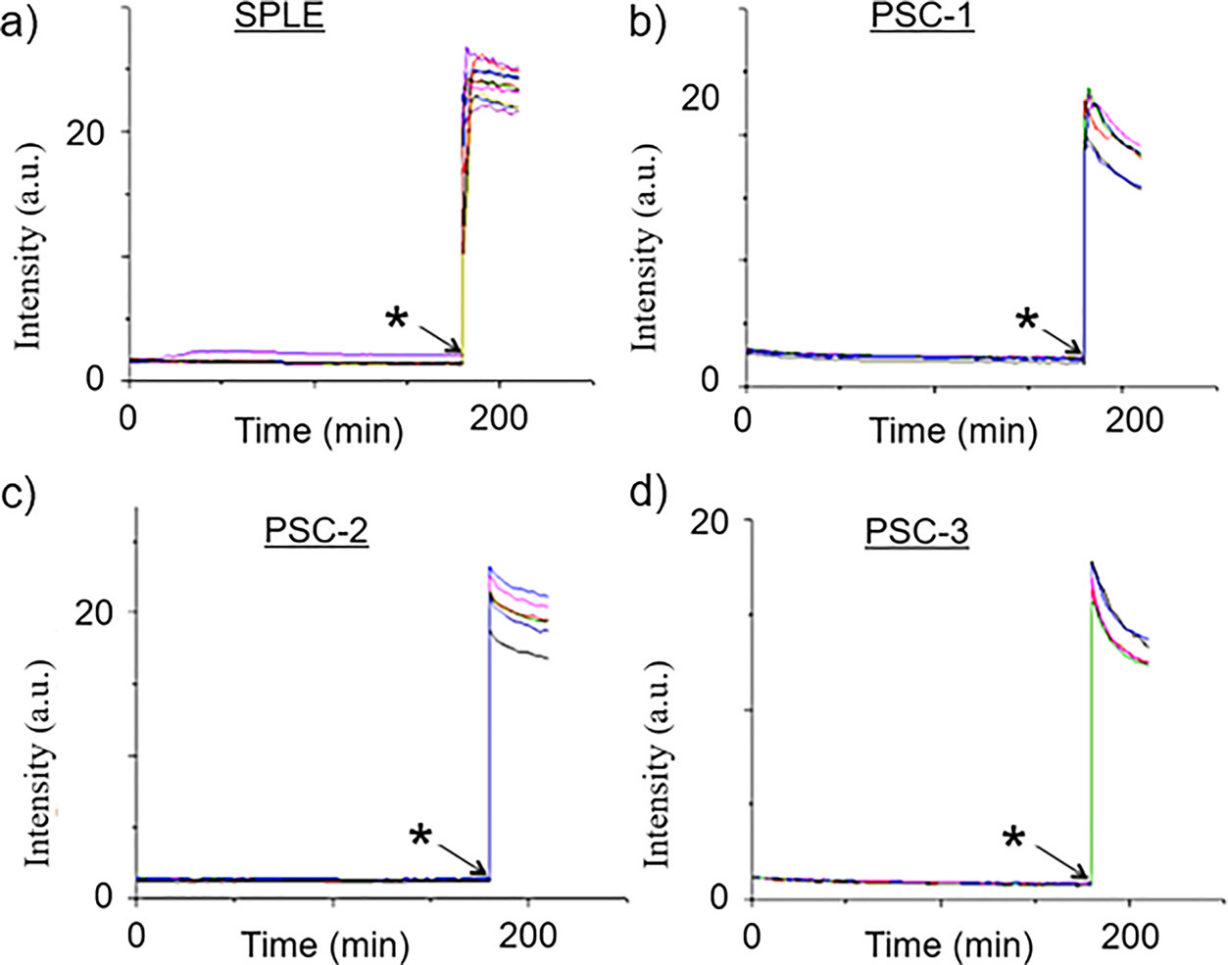
Calcein leakage assay of vesicles engineered from (a) SPLE, (b) PSC-1 (c) PSC-2, and (d) PSC-3 loaded with 50 mM calcein and monitored for 3 hours on a 96 well plate using a fluorescence spectrophotometer. The data shows that no calcein leakage was observed until detergent was added to the system (denoted by the asterisk), indicating that our plant lipid vesicles were both formed successfully and were stable for the duration of our experiments.

We performed a calcein release assay as a control to show that stable and intact unilamellar vesicles could be made from our plant lipid preparations (Fig. 2). Given that calcein leakage from the vesicles is associated with a significant fluorescent signal due to the transition of calcein from the self-quenched to the unquenched state, the absence of any fluorescence for the duration of our 180 min measurements for Figs. 2(a) SPLE, 2(b) PSC-1, 2(c) PSC-2, or 2(d) PSC-3 indicates that stable vesicles were successfully formed for each of our plant lipid formulations. This is also implied in each case by the appearance of a strong fluorescent signal upon releasing the calcein into the external environment, triggered by the addition of detergent to the system (indicated by the asterisk in Fig. 2). Our results show that all of our plant lipid mixtures can be used to make stable vesicles.

### B. Assembly of DIBs and DIB networks from plant lipids

**FIG. 3.**
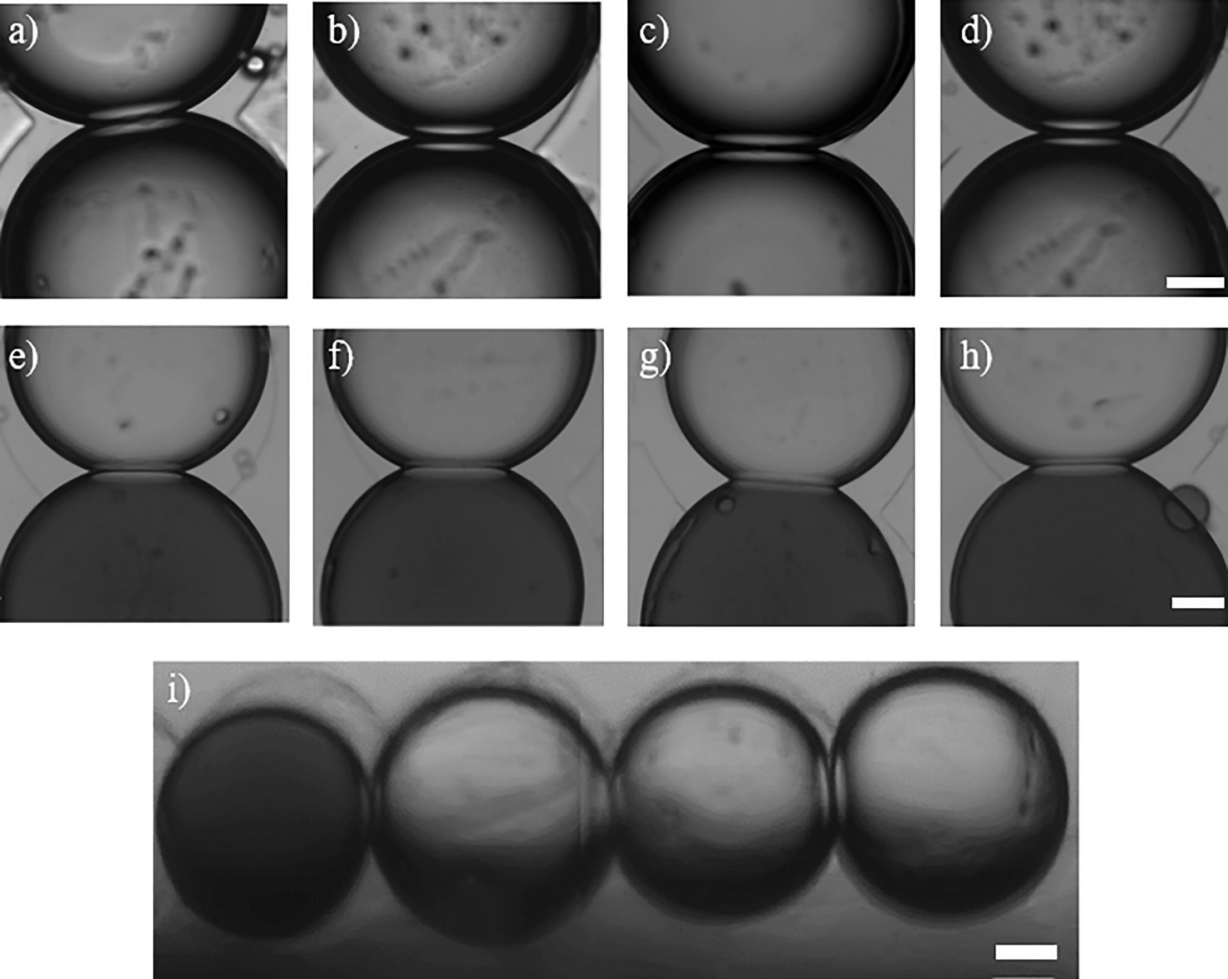
Brightfield micrographs of symmetric and asymmetric DIBs formed from plant lipids. (a)–(d) Symmetric DIBs formed between droplets containing vesicles of (a) SPLE (b) PSC-1, (c) PSC-2, and (d) PSC-3 with interfacial areas of 86, 65, 87, and 53 *μ*m^2^ respectively. (e)–(h) Asymmetric DIBs formed between a droplet containing DOPC vesicles (loaded with calcein) and a second droplet containing (e) SPLE, (f) PSC-1, (g) PSC-2, and (h) PSC-3 vesicles with interfacial areas of 68, 87, 88, and 68 *μ*m^2^. (i) DIB network assembled from one DOPC droplet (left) and three droplets containing SPLE vesicles with interfacial areas of 34, 48, and 48 *μ*m^2^, respectively. All images were obtained 5 min after the droplets were placed into contact. Scale bars = 200 *μ*m.

Compositionally symmetric and asymmetric DIBs made from our plant lipid mixtures are shown in Fig. 3. Brightfield micrographs of symmetric DIBs, where both droplets contained vesicles composed of SPLE, PSC-1, PSC-2, and PSC-3, are shown in parts (a)–(d) of Fig. 3 respectively. In each case, the images show the formation of an interface when the droplets are placed into contact, which is indicative of DIB formation. The same was found when asymmetric DIBs were formed between a droplet containing DOPC (1,2-dioleoyl-sn-glycero-3-phosphocholine) vesicles (loaded with calcein for clarity) and a droplet containing vesicles composed of SPLE, PSC-1, PSC-2, and PSC-3 as shown in parts e–h of Fig. 3, respectively. To our knowledge, these represent the most bio-representative and asymmetric DIBs reported to date and are the first examples of DIBs assembled from model plant lipids. To show that our approach to engineer plant DIBs can also be applied to assemble networks of model plant membranes, we also constructed a linear DIB network (Fig. 3(i)) consisting of a droplet containing DOPC vesicles (left) and three droplets containing SPLE vesicles. The appearance of a defined interface between each droplet implies that the DIB network has successfully formed and indicates that our approach could be used to assemble higher-order networks of model plant bilayers.

### C. Permeation of small molecules across plant DIBs

**FIG. 4.**
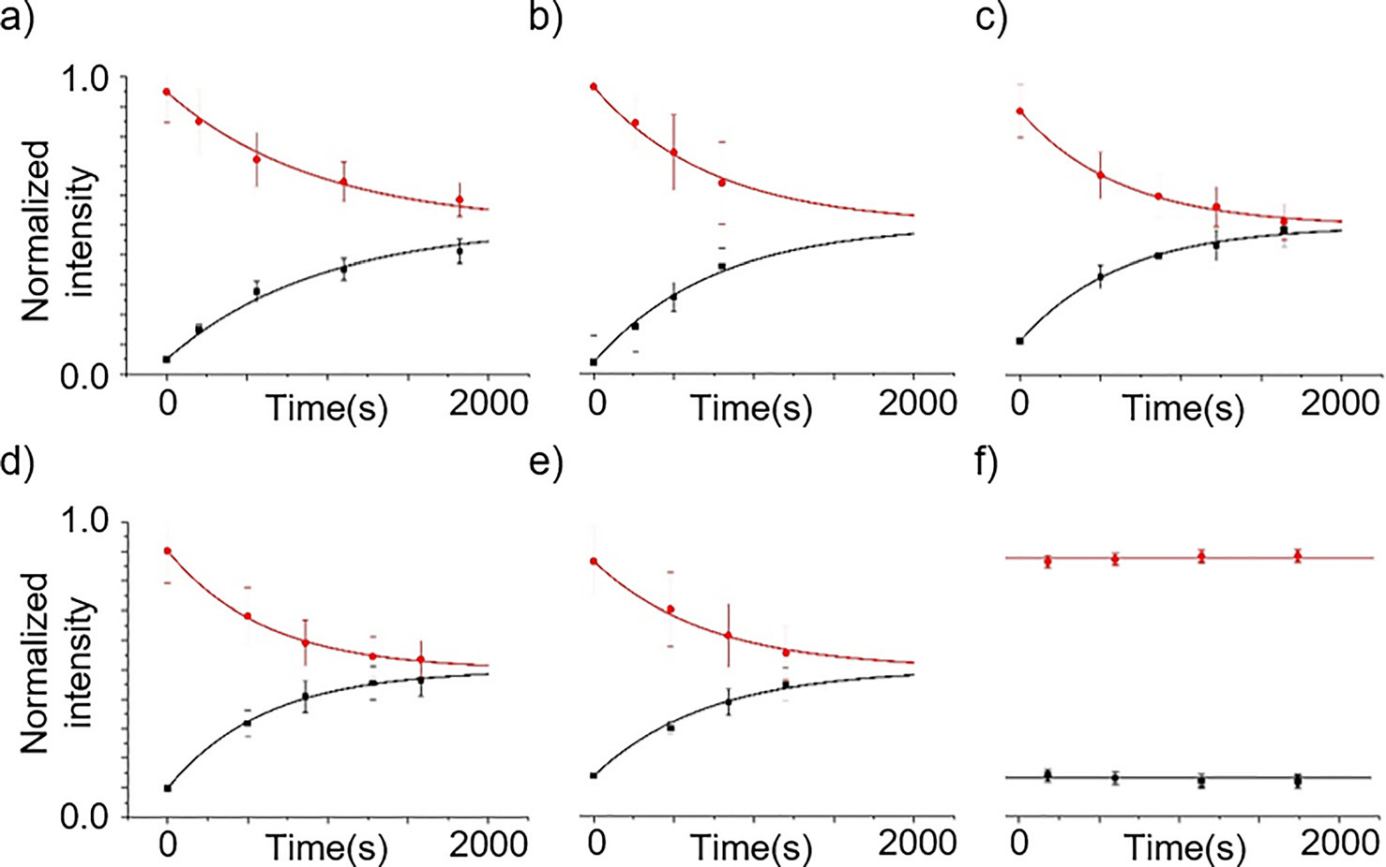
Fluorescence assay investigating bilayer permeability following the formation of asymmetric DIBs from plant lipids. DIBs were formed between a source droplet containing DOPC vesicles and resorufin and a sink droplet containing vesicles made with (a) DOPC (n = 17), (b) SPLE (n = 8), (c) PSC-1 (n = 5), (d) PSC-2 (n = 16), (e) PSC-3 (n = 9), and (f) control (n = 6). The normalized fluorescence intensity is shown in the source (black squares) and sink (red circles), along with their standard deviations. The data show resorufin diffusing out of the source droplet into the sink.

We performed a bilayer permeation assay using the fluorescent and membrane permeable molecule resorufin to confirm that asymmetric DIBs were successfully formed using our plant lipid preparations. Fig. 4 shows the normalized fluorescence intensity of DIBs formed between a “source” droplet containing resorufin and DOPC vesicles, and a “sink” droplet containing vesicles made from either DOPC, SPLE, PSC-1, PSC-2, and PSC-3 in parts (a)–(e) of Fig. 4, respectively. When the droplets were placed into contact, a decrease in the amount of resorufin detected in the source droplet was always associated with an increase in the amount detected in the sink droplet for each condition over time by Equation (1). In contrast, the fluorescence of the droplets remained unchanged when the droplets were placed in near proximity to each other but were not in contact (Fig. 4(f)). This confirms that resorufin is primarily diffusing across the lipid bilayer and not into the surrounding lipid-oil medium.

**FIG. 5.**
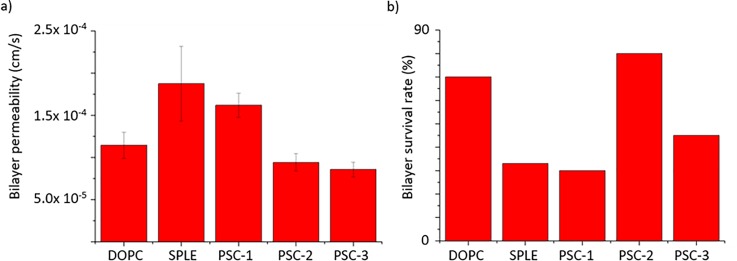
(a) Measurements of effective bilayer permeability values of resorufin across symmetric and asymmetric membranes composed of DOPC and the indicated lipid formulation. The addition of cerebrosides appears to decrease the effective bilayer permeability. (b) Bilayer survival rates of symmetric and asymmetric DIBs composed of DOPC and the indicated lipid composition.

The effective permeability values extracted from our data are summarized in Fig. 5(a), which shows that there was little statistical difference between symmetric DOPC DIBs and asymmetric DIBs composed of DOPC:PSC-2 and DOPC:PSC-3, but a significant increase in the effective permeability of DIBs composed of DOPC:SPLE and DOPC:PSC-1. While previous studies have shown that the addition of cerebrosides^37,38^ or cholesterol^38,39^ reduces the permeability of liposomes, these reports also indicate that cerebrosides have a more potent effect compared to cholesterol, which is in-line with our findings. The presence of unknown components in SPLE makes it difficult to speculate why DOPC:SPLE DIBs were significantly more permeable compared to DOPC:DOPC DIBs, although this could be due to the presence of 22.1% PE which has been shown to increase the permeability to fluconazole in skin membranes.^40^ In any case, our data confirms the presence of asymmetric DIBs made from our plant lipid formulations and clearly shows that our different membrane compositions can give rise to differences in the effective membrane permeability, suggesting that membrane permeability could be specifically tuned to replicate other plant membranes. In order to realize this goal, and to engineer more elaborate higher-order model plant systems, it is essential that the DIBs assembled are stable and do not rapidly coalesce. We found that over 70% of our DOPC:DOPC DIBs and DOPC:PSC-2 DIBs were stable for the duration of our 20 experiments, while survival rates for DOPC:SPLE, DOPC:PSC-1, and DOPC:PSC-3 were less than 50% (Fig. 5(b)). Although there does not appear to be any correlation between bilayer stability and effective bilayer permeability, the addition of cerebrosides and sterols at specific concentrations appears to stabilize the membrane, while it is also of note that the most stable DIBs contained the least SPLE.

## IV. CONCLUSION

In summary, we show the assembly of asymmetric plant DIBs engineered to replicate the plasma membrane composition of *Arabidopsis thaliana*, tobacco, and oats for the first time. Our results indicate that cerebrosides can decrease the effective permeability of model membranes, while the addition of cerebrosides and sterols at specific concentrations could have a stabilizing effect on DIBs. Our DIBs are among the most compositionally complex and bio-representative reported to date, particularly as the phospholipid content of plant membranes is understood to be highly conserved throughout plant growth and reproduction.^41^ Our results represent a significant milestone towards creating a model system capable of mimicking natural translocation pathways found in plants, this combined with a high-throughput approach using droplet microfluidics, and new technologies to address the unstirred water layer limits,^42,43^ could set a new paradigm for screening new herbicides and for probing the translocation profiles of natural molecules and agrochemicals to determine the rules which dictate how physico-chemical and biological variables such as membrane composition, temperature, and drought can impact photosynthetic yield.
